# Progress in Plasmonic Sensors as Monitoring Tools for Aquaculture Quality Control

**DOI:** 10.3390/bios13010090

**Published:** 2023-01-05

**Authors:** Gabriela Elizabeth Quintanilla-Villanueva, Jesús Maldonado, Donato Luna-Moreno, José Manuel Rodríguez-Delgado, Juan Francisco Villarreal-Chiu, Melissa Marlene Rodríguez-Delgado

**Affiliations:** 1 Universidad Autónoma de Nuevo León, Facultad de Ciencias Químicas, Av. Universidad S/N Ciudad Universitaria, San Nicolás de los Garza 66455, Mexico; 2 Centro de Investigación en Biotecnología y Nanotecnología (CIByN), Facultad de Ciencias Químicas, Universidad Autónoma de Nuevo León. Parque de Investigación e Innovación Tecnológica, Km. 10 autopista al Aeropuerto Internacional Mariano Escobedo, Apodaca 66629, Mexico; 3 Department of Neurosurgery, School of Medicine, Yale University, New Haven, CT 06510, USA; 4 Centro de Investigaciones en Óptica AC, Div. de Fotónica, Loma del Bosque 115, Col. Lomas del Campestre, León 37150, Mexico; 5 Tecnológico de Monterrey, School of Engineering and Sciences, Av. Eugenio Garza Sada Sur No. 2501, Col. Tecnológico, Monterrey 64849, Mexico

**Keywords:** plasmonic sensor, biosensor, aquaculture, SPR, multiplex detection

## Abstract

Aquaculture is an expanding economic sector that nourishes the world’s growing population due to its nutritional significance over the years as a source of high-quality proteins. However, it has faced severe challenges due to significant cases of environmental pollution, pathogen outbreaks, and the lack of traceability that guarantees the quality assurance of its products. Such context has prompted many researchers to work on the development of novel, affordable, and reliable technologies, many based on nanophotonic sensing methodologies. These emerging technologies, such as surface plasmon resonance (SPR), localised SPR (LSPR), and fibre-optic SPR (FO-SPR) systems, overcome many of the drawbacks of conventional analytical tools in terms of portability, reagent and solvent use, and the simplicity of sample pre-treatments, which would benefit a more sustainable and profitable aquaculture. To highlight the current progress made in these technologies that would allow them to be transferred for implementation in the field, along with the lag with respect to the most cutting-edge plasmonic sensing, this review provides a variety of information on recent advances in these emerging methodologies that can be used to comprehensively monitor the various operations involving the different commercial stages of farmed aquaculture. For example, to detect environmental hazards, track fish health through biochemical indicators, and monitor disease and biosecurity of fish meat products. Furthermore, it highlights the critical issues associated with these technologies, how to integrate them into farming facilities, and the challenges and prospects of developing plasmonic-based sensors for aquaculture.

## 1. Introduction

Nowadays, aquaculture is the fastest-growing food-producing sector in the world, providing about 17% of animal proteins and 7% of all proteins globally by 2019 [1]. This economic activity, referred to as farming aquatic organisms (e.g., fish, molluscs, and crustaceans), contributed in 2020 to the global production of 122.6 million tonnes, worth USD 281.3 billion [1]. Aquaculture activities are practised inland, in coastal or marine environments in various facilities, from ponds and cages to highly sophisticated water reuse systems [2]. In particular, the expansion of aquaculture showed a boost during the late 1980s to 2020, showing a growth in the production of aquatic organisms in inland waters, from 12% to 37%. Meanwhile, it is forecast that by 2030, aquatic food production will increase by a further 15% [1].

However, this growth requires developing and adopting innovative technologies for more efficient and resilient aquaculture. In addition, this industry faces various challenges that complicate its operation [3]. For example, maintaining good water quality throughout the culture is a crucial challenge due to self-pollution by inorganic nutrients, food remnants, and fish faeces, as it causes eutrophication in the surrounding environment due to the high nutrient stimulus [3]. On the other hand, there is the additional challenge of controlling the growth of pathogens, which are directly involved in the infection of the fish, which causes significant loss of profits in the industry, not to mention the risk of generating resistance to antibiotics due to the use of conventional control antimicrobials [4]. Finally, farmed fishes are commonly reared at large scales in high densities, which causes stress and significantly increases the mortality of animals [5].

**Figure 1:**
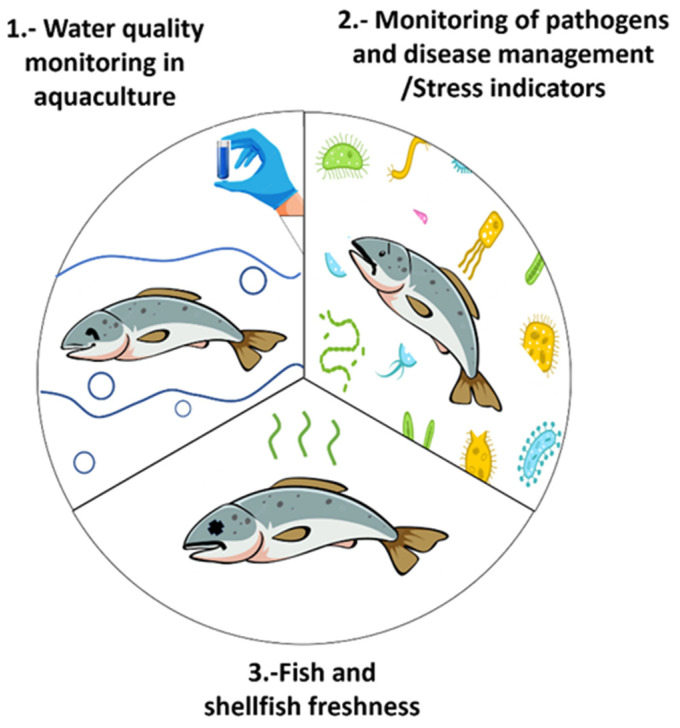
Major areas of interest in aquaculture monitoring.

Despite these challenges, aquatic food producers are responsible for ensuring and providing consumers with fresh and safe products. To do so, companies must demonstrate the absence of hazardous compounds in their products. Unfortunately, this is no easy task, as toxin analysis currently takes 24 h up to days from the point of sample to obtain a result. [5]. Therefore, aquaculture operators must conduct their real-time end-product testing for regulatory acceptance to ascertain the safety and release of their products. Consequently, the European market for analytical tests developed for food safety applications, especially for pathogen detection, has grown to an estimated $4 billion by 2018 [6], showing the sector’s economic importance in detecting methods for monitoring each central area in aquaculture systems. In this sense, there is currently a broad range of analytical methods for the concentration assessment of crucial chemical compounds for aquaculture safety, including spectrophotometric, chromatographic, and fluorometric techniques, and electrochemical analysis, among others [7]. Mainly, photonic sensors have gained particular interest as they enable online and continuous monitoring, suitable for in situ and in vivo measurements, making them very advantageous for aquaculture systems. Figure 1 outlines the current plasmonic sensing technologies trialled and applied at the aquaculture process’s main stages, from the species’ farming up to their harvest and previous distribution. The purpose of this review is to (i) discuss the recent advancements in plasmonic technologies, including conventional prism and fibre-based SPR sensing, as well as nanoparticles localise SPR, and describe their drawbacks, (ii) illustrate the application of these sensors for monitoring the aquaculture operations in fish farms, detection of environmental hazards, and tracking fish health by biochemical indicators, and (iii) highlight the current challenges and the future perspectives of developing plasmonic-based sensors in this field. Therefore, the paper’s architecture is organised as follows: Section 2 of the paper presents an overview of the operating principles of plasmonic-based sensors. Section 3 describes the types of recognition elements employed in sensing applications and the assay formats. Section 4 describes in detail the use of plasmonic sensors in aquaculture operations to monitor the quality of tanks’ water, detecting nitrogenous compounds, micropollutants, and stress indicators. Section 5 and Section 6 discuss the significant findings in plasmonic-based sensors for monitoring pathogens and harmful algal bloom toxins. Section 7 gives an overview of the plasmon sensing techniques to evaluate the freshness of fish and shellfish by detecting biogenic amines. Finally, Section 8 presents the final remarks and perspectives.

## 2. Optical Sensors Based on Plasmonic Techniques

Plasmonic sensors are based on an optical phenomenon generated by incident polarised light between a dielectric and a metallic system, forming an evanescent field from the waving electrons that propagate along the system, known as surface plasmon waves (SPW) [5]. Some of the sensors based on the plasmonic technique are surface plasmon resonance (SPR), localised SPR (LSPR), and fibre-optic SPR (FO-SPR). These sensors are commonly based on noble metallic thin films and nanostructures (gold, silver, platinum, and palladium) due to their property of a higher optical absorption band in the visible–near infrared range, called a plasmonic band [8]. Moreover, the depth and position of the SPR dip are sensitive to the refractive index and employed as analytical parameters in a wide range of applications. Furthermore, plasmonic sensors possess advantages compared to conventional technologies, which include detection with no labels needed, high sensitivity, and real-time analysis. However, they suffer some drawbacks, such as the condition of a p-polarised light to induce the optical phenomenon, the requirement of recognition elements to offset its low selectivity, and a range of RI changes detection around 200 nm [9]. Table 1 summarises some of the advantages and disadvantages of plasmonic sensors compared to other technologies employed in aquaculture applications.

**Figure 2:**
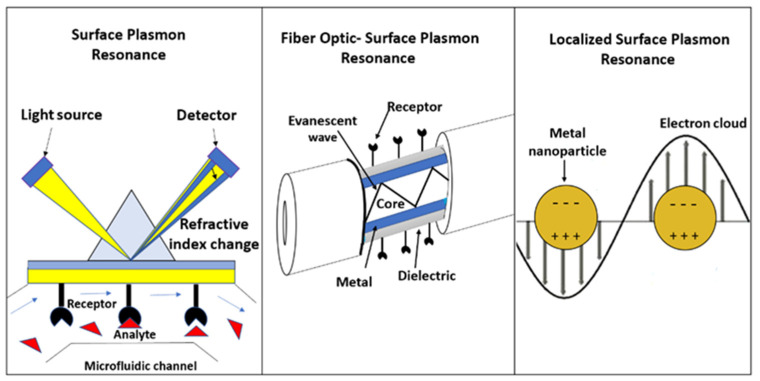
Schematic representation of prism-based surface plasmon resonance, fibre-optic SPR, and localised SPR.

Plasmonic sensors can be presented in different configurations. For example, SPR-prism-based sensors utilise a thin metal film between two transparent media, a glass prism, and a sample solution. The polarised light enters the glass prism and undergoes total internal refraction (above a critical angle of incidence), allowing the evanescent wave to penetrate the gold film [8]. Polarised light is necessary for the excitation of the surface electrons of the noble metal to occur and, thus, the phenomenon of surface plasmon resonance. This light must have a p-polarisation (parallel to the plane of incidence), otherwise, the phenomenon will not happen. S-polarisation (perpendicular to the plane of incidence) cannot excite surface electrons [18]. Thus, the interaction of the evanescent wave and resonating electrons at the gold film surface will cause the excitation of surface plasmons, decreasing the reflected light intensity (the phenomenon of surface plasmon resonance). SPR is observed as a sharp dip in reflected intensity at a certain angle, which shifts when biomolecules bind to the surface, changing the refractive index of the surface (Figure 2).

On the other hand, the optical fibre configuration is also used to implement SPR (FO-SPR), shown in Figure 2. In this sensor, the fibre cladding is removed, leaving the bare core exposed, and coated with a metal film layer to create the sensing region [19]. The sensing length influences the reflections of the light beam in the core, related to the width of the SPR [20]. Under this configuration, a range of guided rays is launched into the fibre, generating the evanescent field by total internal reflection, which excites surface plasmons between the dielectric and metallic system [11]. The wavelength at which the dip occurs by the SPR phenomenon is the resonance wavelength and shifts as the refractive index change (spectral wavelength interrogation) [11]. The fibre-optic sensor can be used in two modes: transmissive and reflective. In the transmissive mode, the sensing region is in the middle of the fibre. The light is coupled from the source to the fibre through one end, and the analyser is connected to the other end [21]. In the reflective configuration, the sensing region (metal coating) is located at one end of the fibre, reflecting the incident light as a result of the mirror effect of the metal coating [22].

In terms of metallic nanostructures, when a light beam incident gets trapped, it causes the waving of dislocated electrons, resulting in the localised surface plasmon effect (LSPR), schematically shown in Figure 2. In this case, the resonance wavelength depends on the shape and size of the nanomaterial, as well as the medium surrounding it [19]. For LSPR, the wavelength interrogation method allows the evaluation of changes in absorbance wavelength caused by the changes in the refractive index [23]. The main disadvantage of LSPR is its lower refractive index sensitivity compared to conventional SPR, which is compensated by its high sensing surface for monitoring binding events between the surface and a single analyte [23]. The sensitivity of plasmonic nanoparticles is dependent on their size and geometry. For example, nanostructures with intense absorption or scattering properties are closely related to the increasing size of gold nanoparticles. Small-size particles (∼40 nm) are preferred for high-absorption cross-sections. Meanwhile, a dominant scattering property will be observed in relatively big-size nanoparticles (∼80 nm) [24].

Regarding nanoparticle geometries, previous reports have established that anisotropic structures show higher sensitivity than spherical-shaped structures [25,26]. For instance, nanorods, nanocubes, nanoshells, and nanoholes have been studied, showing higher yields than conventional spheres [24]. In this sense, gold nanorods present a refractive index sensitivity of ∼250 nm compared to ∼60 nm for gold spheres [27]. Meanwhile, exotic shapes such as trimmers and nanopillars have been reported to provide adjustable wavelengths from ultraviolet to near-infrared, enhancing the LSPR sensitivity up to 675 nm/refractive index units. Moreover, it allows sharp resonances and a well-localised electromagnetic field that improves plasmonic sensing properties [28]. In addition, plasmonic geometries with chiral properties have been reviewed, reporting the improvement of light-adjusting interactions [29]. For example, twisted, gammadion, or shuriken 3D structures have been reported to allow the change of circular dichroism of molecules from ultraviolet to the visible region, simplifying the analysis in sensing applications [29].

On the other hand, LSPR-based sensors have also benefited by integrating semiconductor particles called quantum dots (QDs) [30]. These particles (commonly up to 10 nm) emit extended fluorescence when excited by light. The QDs are usually based on carbon, silicon, cadmium, or indium complexes [31]. Due to QDs having been extensively used as fluorescent labels, in combination with the surface plasmon properties of gold nanoparticles, they can enhance the sensitivity of plasmonic detection systems [32]. When they are adjacent to metallic nanoparticles they can affect the fluorescence signal and, therefore, be quenched depending on the analyte amount [33].

Despite the progress on plasmonic materials, geometries, and set-up configurations [34], little has been applied to aquaculture activities, leading to broad development perspectives. Regardless of the type of plasmonic sensor or their configuration scheme, their performance is still subject to modifying the metallic surface (functionalisation) with binders or ligands to provide specificity and selectivity to the monitoring system.

## 3. Recognition Elements and Assay Formats in Plasmonic Sensors

**Figure 3:**
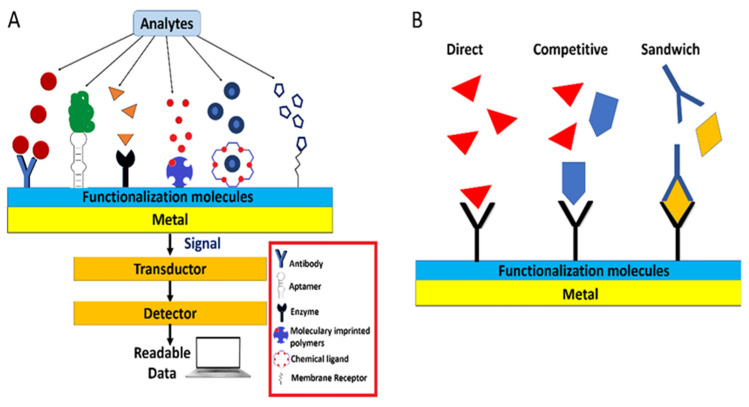
(**A**) Schematic representation of types of recognition elements and (**B**) Assay formats.

The correct choice of a binding partner or ligand can improve the detection analysis, avoiding non-specific attachments and cross-reactivity of other chemical compounds in the sample. Therefore, a successful binder should be a molecule that recognises the analyte with excellent specificity and high affinity [35] (see Figure 3A). Several types of binder have been exploited in developing plasmonic sensors in aquaculture applications.

For example, membrane receptors can be referred to as cell-membrane-bound chemical structures or cytoplasmic proteins exploited for their ligand-binding capabilities. In this sense, the saxiphilin receptor, presented in various amphibians and terrestrial invertebrates, has been studied as a binder to paralytic shellfish poisons detection by SPR and is a toxin of great importance to detect in seafood [36,37].

On the other hand, antibodies are Y-shaped biochemical binders (immunoglobulins) produced by B cells as a natural adaptative response to foreign molecules. In particular, they are widely used in research to detect target proteins or a chemical moiety on the structure of molecules of interest. The recognition is based on a specific antigen’s amino acid sequence [38]. Polyclonal antibodies contain a heterologous mixture of immunoglobulins against the whole antigen. In contrast, monoclonal antibodies are composed of identical immunoglobulins against one epitope, a specific amino acid sequence in the protein recognised by the antibody [35]. In this sense, several studies have employed antibodies as a recognition element in aquaculture applications, such as toxin detection [39], water quality control in fish ponds [40,41], and pathogen monitoring.

Also, enzymes are very attractive recognition biomolecules employed in plasmonic sensors due to their various measurable catalytic reactions towards specific molecules, producing conformational changes, electrons transfer, and heat, among others [38]. For example, a study established the quantification of yessotoxin, a seafood toxin, through a direct assay, where the interaction of the toxin with the enzyme phosphodiesterase was observed using SPR equipment [42].

Furthermore, recent advances in the development of sensing devices have broadened the horizon to design synthetic receptors, unlike the previously mentioned biomolecules. In this sense, chemical binders or artificial receptors are designed molecules capable of selectively binding analytes through non-covalent interactions. Some examples of these molecular structures are polyalcohols, crown ethers, calixarenes, helicenes, sterically geared tripods, metal complexes, pinwheels, porphyrins, and fused ring heterocycles. In this sense, Chen et al. employed a crown-like ether, calix [4] arene, as an alternative receptor for saxitoxin detection in shellfish samples [43].

Other artificially created ligands are molecular imprinted polymers (MIPs), where crosslinking monomers are polymerised around the target molecule, forming a template with functionalised groups that mimic the structure of interest [19]. The three-dimensional network of the imprinted sites of the template would be complementary in shape and size to the target molecule, which makes them highly selective [19]. The binding between MIPs and the analyte occurs through non-covalent interactions or reversible covalent bonds. Its applicability has been demonstrated in detecting domoic acid toxin by SPR [44].

Finally, aptamers are alternative binders based on single-stranded nucleic acid that selectively binds with high affinity to a broad range of targets (toxins, whole cells, proteins, etc.) [45]. Aptamers can be created from polymers of nucleic acids (RNA and DNA) or amino acids (peptides) by the well-known method of systematic evolution of ligands by exponential enrichment (SELEX) [46].

However, not only is the recognition element selection crucial for designing and implementing the sensing method, but the assay format is also. The schemes range from the direct, competitive, and sandwich (non-competitive) assay. The detection reaction occurs directly in the assay between the recognition element and the target molecule [47].

A competitive binding assay is based on the competition of an analogue of the analyte (commonly a protein conjugate) versus the analyte in the sample for the receptor binding sites. As the analyte concentration in the sample increases, less analogue can bind to the recognition element, so the measured response decreases. Thus, the response obtained is inversely related to the amount of analyte in the sample, so the lower the signal, the more analyte [35]. This assay approach is often used to analyse small molecules and can be designed under two schemes. In surface competition, protein conjugate is used as a competitor for binding to immobilised receptors. Meanwhile, the solution competition mode is based on mixing a known amount of detecting molecule with the analyte, and the free detecting molecule remaining in the solution is measured [47] (Figure 3B).

In the sandwich assay, the analyte is ‘sandwiched’ between the two specific recognition elements (antibodies). The scheme is based on the immobilisation of the capture molecule (a highly specific antibody), which binds to the analyte when the sample is added, followed by the addition of a second antibody (detection molecule) that binds to an additional segment of the analyte (See Figure 3B). In this case, the signal generated would correspond to the amount of analyte in the sample. This assay is precise since two recognition elements are required [47].

As can be seen, the selectivity of plasmonic sensors can be very high, depending on the type of receptor. For example, immunological assays provide high selectivity because antibodies have specific interactions with the antigen. No matrix effects were observed, even in studies such as that of Mauriz et al. (2006) [48]. In the case of enzyme receptors, the selectivity depends on the assay conditions, but a proper selection of the enzyme is also critical. For example, Li et al. (2002) [49] demonstrated a high selectivity by analysing a sample with a mixture of molecules besides the analyte. On the other hand, synthetic receptors such as aptamers and molecularly imprinted polymers provide remarkable selectivity since these molecules are “custom-designed,” interacting specifically with the target molecule [50].

Thus, to provide an efficient and reliable detection method, it is essential to consider the sensing technique, the recognition molecule, and the type of assay. These are crucial factors that help guarantee its successful implementation to provide an efficient and reliable detection method. Moreover, achieving a low noise level and high stability of the plasmonic sensor depends on a good selection of working conditions. For example, evaluating different buffers and pH could provide the best conditions to allow the highest stability of the receptor [49]. In addition, using highly specific receptors, such as antibodies, provides high selectivity, so the sample matrix does not generate significant interference, avoiding non-specific unions that may yield false positives [51]. 

In this context, the importance of appropriate detection protocols at critical steps during aquaculture practices has prompted many researchers to develop affordable and reliable alternatives based on novel nanophotonic sensing technology. Thus, this study attempts to summarise recent advances in innovative plasmonic sensors (SPR, LSPR, and FO-SPR) tested for the detection of several molecules of interest at different aquaculture stages, from farming the aquatic organisms to the harvesting process.

## 4. Aquaculture Operations and Water Quality Monitoring

For the smooth operation of aquaculture systems, it is crucial to maintain specific parameters in concentrations within acceptable limits to avoid adverse conditions that could affect the fish hatchery’s growth and survival. The most critical water quality parameters in aquaculture activities are temperature, salinity, pH, dissolved oxygen, ammonia (NH_3_), nitrite (NO_2_^−^), and nitrate (NO_3_^−^) [52].

### 4.1. Monitoring of Nitrogenous Compounds

The presence of nitrogenous compounds is commonly associated with the leaching of fertilisers to farming ponds, fish excretion, or decomposition of uneaten feed. In aquifers, inorganic nitrogen is mainly in the form of nitrate (NO_3_^−^) and ammonium (NH_4_^+^) ions. However, due to the aerobic process of nitrification, ammonium tends to be oxidised to nitrate (NO_3_^−^). At the same time, nitrite (NO_2_^−^) is an intermediate product in the nitrification process [52]. According to the study of Zhou and Boyd (2016), the average values of total ammonium nitrogen concentration (sum of NH_3_-N and NH_4_^+^-N) found in a commercial marine recirculating aquaculture system (RAS) for the culture of sea bass (*Dicentrarchus labrax*) and sea bream (*Sparus aurata*) ranged from 0.06 to 6.56 mg L^−1^ of N. Regarding nitrite and nitrate, concentrations ranged from 0.10 to 3.37 mg L^−1^ and 25.10 to 62.77 mg L^−1^, respectively, being 46 mg L^−1^ of NO_3_^−^N, the maximum allowable level required in the hatchery [52]. High levels above 100 mg L^−1^ of NO_3_^−^ and 2 mg L^−1^ of NO_2_^−^ negatively impact the aquaculture system [53].

In this sense, the surface plasmon resonance imaging (SPRi) method has already been employed in water sensing NO_3_^−^ and NH_4_^+^. Bioactive chips were designed by immobilising nitrate reductase from *Aspergillus niger* and glutamine synthetase from *Escherichia coli* on gold-coated chips to allow biorecognition. The sensor was applied in water samples, diluted in a buffer before the measurement, where NO_3_^−^ and NH_4_^+^ ranged from 24 to 780 mg L^−1^ and 0.26 to 120 mg L^−1^, respectively [54]. Meanwhile, Miao et al. [55] developed an SPR-based NO_2_^−^ with a detection limit of 3.0 µg L^−^^1^. The nanosensor combined surface-modified gold nanoparticles (AuNPs) with a colourimetric assay.

Regarding ammonia (NH_3_), its presence in fish farms at chronic levels may reduce the appetite and growth of the species, eventually increasing its mortality [56]. Depending on the fish species and life stages, the tolerance to NH_3_ may vary. Thus, the Food and Agriculture Organization (FAO) has established that NH_3_ is toxic at levels above 0.02 mg L^−1^ [53]. Optical fibre sensors have been used to detect this analyte, based on the interaction of the decorated probe with nanoparticles, nanocomposites or dyers, and the dissolved NH_3_, resulting in changes of intensity [57] or wavelength [58]. Mohammed et al. proposed a single-mode fibre coated with polyaniline (PANI)/graphite nanocomposite to detect NH_3_ at concentrations as low as 14 mg L^−1^ [58], higher than the value established by the FAO [53].

On the other hand, optical fibres have also been modified with polymers to allow their use in NH_3_ detection, for example, a polymeric optical fibre using Oxazine 170 perchlorate as a sensing material. As a result, measured differences in the fluorescence of 7-amino-4-trifluoromethyl coumarin (AFC) were recorded as a function of the ammonia levels due to the absorption change of the sensing material [59]. Meanwhile, Jalal et al. (2012) tested a clad-modified optical fibre with Oxazine 170 perchlorate in stagnated and dynamic water [57]. In addition, a thin film of gold/palladium was evaporated onto a fibre end, acting as a reflector for the optical signals, allowing it to work in reflection mode, which resulted in a better performance for the NH_3_ detection in high-salinity water, where the concentrations were as low as 100 µg L^−^^1^ [60]. In general, the optical sensing probes provide operational ease for water-monitoring applications. Furthermore, they allow being readily introduced into the tanks, permitting the remote operation of appliances along the optical fibres and even eliminating the need to take samples to be analysed in external equipment, making this type of sensor very suitable for aquaculture [60].

### 4.2. Monitoring of Biocides and Micropollutants

Currently, the increasing expansion of the aquaculture industry requires more frequent disinfectants and antifoulant compounds to control the growth of harmful microorganisms in aquaculture facilities, improve survival rates, and control pathogens and diseases in hatcheries [61].

However, uncontrolled concentrations of these chemicals may also deter the fish and shellfish health. Ingestion of the contaminants could also result in lower growth rates or death of cultured fishes, posing a significant risk to human health by ingestion [62,63]. Nevertheless, the aquaculture farms’ location plays a crucial role in the contamination by micropollutants, such as herbicides, which have been detected in fish farms, spread through aerosol or runoffs from adjacent croplands [64,65,66]. For example, Naessens et al. developed a fibre-optic biosensor using *Chlorella vulgaris* to determine herbicides such as atrazine, simazine, and diuron [61]. The microalgae (*C. vulgaris*) were employed as a biorecognition element by its immobilisation on membranes placed onto the tip of the fibre. The biosensor was based on kinetic measurements of chlorophyll-a fluorescence in the presence of the herbicides, which affected the photosynthesis process of *C. vulgaris* [61]. Agrawal et al. proposed a detection method for atrazine using an unclad optical fibre covered with a 40 nm thick Ag layer and a molecularly imprinted polymer (MIP) as a template-specific recognition site. The MIP layer was prepared from monomers of methacrylic acid (MAA) and 2-hydroxymethacrylate (HEMA), showing a limit of detection of 1.92 × 10^−14^ M [67].

Meanwhile, Chacorro-Ruiz et al. developed an interferometric nanobiosensor for the label-free detection of the biocide Irgarol 1051 in seawater through a competitive inhibition immunoassay, where the signal obtained is inversely proportional to the concentration of the contaminant in the sample [40]. The biosensor showed a limit of detection of 3 ng L^−1^ without requiring sample pre-treatments and reusability during 30 assay-regeneration cycles [40]. Also, an integrated asymmetric Mach–Zehnder interferometer was employed as a multiplexed platform for Irgarol 1051 and tetracycline analysis. Both pollutants were detected using a competitive immunoassay scheme, obtaining a limit of detection for tetracycline and Irgarol 1051 of 0.04 μg L^−1^ and 0.07 μg L^−1^, respectively. The nano-immunosensor was integrated into a buoy to perform the measurements in natural conditions, demonstrating a reliable platform [68]. Another multiplexed system was reported by Yazdi et al. (2013), accomplishing the detection of three highly controlled aquaculture fungicides: methyl parathion (5 mg L^−1^), malachite green (0.1 μg L^−1^), and thiram (5 μg L^−1^) [69]. The optofluidic SERS system comprehends two multimode fibre-optic cables inserted into polydimethylsiloxane microchannels and aligned to the detection zone. The detection zone consisted of a porous matrix packed with silver nanoparticles and adsorbed fungicide molecules [69].

An integrated optical surface plasmon resonance immunoprobe was employed to detect herbicide simazine in the aqueous environment through a binding inhibition immunoassay. The detection limit was 0.16 μg L^−1^ using anti-simazine IgG antibodies and 0.11μg L^−1^ using anti-simazine Fab fragments [41]. Meanwhile, the analysis of the pesticide carbaryl in natural water samples was accomplished using an SPR immunosensor based on a binding inhibition scheme in which the carbaryl conjugate was immobilised onto the gold-coated chip. The reusability of the platform was 220 regeneration cycles, with a limit of detection of 1.38 µg L^−1^. Matrix effects were also analysed in different water sources, such as river water and groundwater, showing no matrix effects when the samples were measured directly and without any sample treatment [48].

### 4.3. Monitoring of Fish Health by Stress Indicators

In aquaculture, it is expected that a high density of fish per pond is maintained to increase production efficiency, leading to a deterioration of water quality. The accumulation of residual bait excrement, ammonia, and nitrous acid, as well as some physical factors such as flow velocity and temperature, are some elements that tend to induce stress in the organisms, increasing their probability of illness [70]. Thus, the aquatic species’ welfare is highly related to the water quality. When they are in contact with contaminated water for long-term periods, they tend to increase their cortisol, urea, and creatinine levels as a response [49]. Assessing the stress levels of organisms in aquaculture, directly in the water, is of utmost importance to understand its interaction with their welfare [5]. In this context, various biosensors have been developed to improve the health checks of cultured species.

The cortisol concentration range in water fish tanks is roughly between 0.007 and 5 ng mL^−1^, depending on the stocking density. However, the lack of technologies for in situ monitoring makes difficult the detection of this critical parameter. In addition, the standard protocol to detect cortisol in fish is by blood analysis, which results may be doubtful since it is an invasive and stressful method [5].

Scarce information on steroid accumulation and levels in aquatic species farms or recirculating aquaculture systems (RAS) is available. Mota et al. (2014) determined the variation of cortisol and sex hormones in RAS, showing steroid concentrations in the rearing effluent ranged between: 3.8–217.0 ng L^−1^ for cortisol, 3–12.5 ng L^−1^ for testosterone, and 0.9–7.1 ng L^−1^ for 11-ketotestosterone [71]. These results suggest that the augmentation of fish production through decreased make-up water use will lead to an accumulation of steroids in the water [71]. In addition, the influence of a high loading density during carp (*Cyprinus carpio*) growth was investigated, showing high cortisol levels in the water as the density increased. Therefore, stress monitoring was indirectly allowed by cortisol measurements [72].

In this context, a polymer optical fibre decorated with gold/palladium (AuPd) amalgam was also used for cortisol detection. In the study, the fibres were modified with anti-cortisol antibodies and tested against cortisol ranging from 0.005 to 10 ng mL^−1^. The detection limit was determined to be 1 pg mL^−1^ [5]. Then, Soares et al. (2022) characterised an FO-SPR immunosensor with a D-shaped geometry for cortisol detection. The functionalised immunosensor with anti-cortisol was tested in concentrations of 0.01 to 100 ng mL^−1^ of cortisol. Then, the specificity of the immunosensor was proved against glucose and cholesterol as interferents, obtaining a sensitivity of 0.65 ± 0.02 ng mL^−1^ with a limit of detection (LOD) of 1.46 ng mL^−1^ [73].

Furthermore, Li et al. reported using an optical probe to detect creatinine in fish farming water, showing a sensitivity and limit of detection of 3.1 and 86.12 µM, respectively [49]. The probe was functionalised with gold nanoparticles (LSPR), niobium carbide, MXene, and creatinase enzyme, tested in a concentration range of 0-2000 µM. Finally, the selectivity of the probe was tested in the presence of interferences such as creatine, sarcosine, ascorbic acid, pyruvate acid, and uric acid. The results showed the most significant wavelength shift in the presence of creatinine compared to the other molecules [49].

On the other hand, a six-channel homemade SPR biosensor was employed to develop a competition assay through monoclonal anti-cortisol molecules, obtaining a detection limit of 0.36 ng mL^−1^ (1.0 nM). The system was tested in saliva, based on simple diffusion through a filter as sample pre-treatments. A detection limit of 1.0 ng mL^−1^ (3.6 nM) was obtained, proving an important approach to a wide range of applications in complex matrixes like aquaculture effluents [74].

## 5. Monitoring of Pathogens and Disease Management

In the aquaculture industry, one of the most significant difficulties is controlling pathogen spreading among aquatic species since the rate of diseases among the species being bred results in significant stock losses due to the mortality index [75]. In addition, they damage muscular tissues or cells during the infection and generate toxins, spoiling the fish, seafood, and related products [76]. Furthermore, early diagnostic methods are crucial for disease management to avoid improper usage of drugs that deposit in the organism’s tissue, discharge into the surrounding water, or cause drug resistance [76]. Thus, an important step is early detection by routine screening under field conditions.

### 5.1. Pathogen Detection

Among bacteria pathogens, *Vibrio* genera, a gram-negative bacteria, is the cause of mass death in cultured fish, shrimps, and shellfish [77,78]. Vibriosis is a disease marked by infection of skin and organs, spread by *V. vulnificus, V. harveyi, V. anguillarum, V. alginolyticus, V. parahaemolyticus,* and *V. salmonicida* [78].

In this sense, an aptamer-based SPR sensor was employed to screen Vibrio parahaemolyticus, obtaining a high selectivity of the aptamer compared to *E. coli, L. monocytogenes, V. fischeri,* and *S. soneii* [79]. Meanwhile, a fibre-optic SPR (FOSPR) biosensor was designed to detect nervous necrosis virus (NNV) in water from grouper farming ponds. The fibre optic was modified by gold nanoparticles towards NNV coat proteins, allowing the pathogen detection at the early infection stage and showing a detection limit of 100 µg L^−1^ [80].

Regarding virus pathogens, plasmonic sensors have also been applied for detection. For example, Lei et al. describe the detection of the white spot syndrome virus (WSSV) by an SPR device based on gold films prepared by gold nanoparticles adsorbed on glass slides (electroless plating) [81]. The white spot disease is a lethal infection in shrimps caused by WSSV. It is characterised by white spots of deposited salts of calcium on the carapace, changes in the body colour (pale or reddish), and lethargy [82]. In the study, antibodies (anti-WSSV) were immobilised on the gold films by self-assembled alkanethiol monolayers, which allowed the concentration of WSSV 2.5 µg L^−1^ in 2% shrimp hemolymph matrix [81]. Later, in 2014, Mai-Ngam et al. developed an SPR method using mixed surfactant chips to detect yellowhead viruses. This pathogen agent also causes mortalities in cultured shrimp. The work established the layer formation of non-covalently bimodal and monomodal dextran chains on an SPR chip, proposing a model of the mixed surfactant matrix layer based on AFM images [83].

### 5.2. Monitoring of Antibiotic Residues Due to Disease Control in Aquaculture

Despite the use of antibiotics, it is a routine practice to prevent or treat diseases in aquaculture systems. However, these compounds’ residues may accumulate in the edible tissues of the treated species or favour antibiotic resistance in pathogens. Thus, several optical devices have been reported to control the incidence of antibiotic contamination in aquaculture production. For example, an SPR-based biosensor has been employed for the selective detection of ciprofloxacin [84] and erythromycin [85] in water. Molecularly imprinted nanoparticles were prepared through a mini-emulsion polymerisation method with methacrylic acid as a functional monomer. The monomer’s surface acted as ciprofloxacin and erythromycin receptors. Then, the nanoparticles were immobilised onto a gold-coated chip. The method showed a limit of detection of 7.1 µg L^−1^ [84] and 290 µg L^−1^ [85] for ciprofloxacin and erythromycin, respectively. Erythromycin was also detected using a fibre-optic core coated with silver and erythromycin-imprinted nanoparticles [86].

On the other hand, the detection of ciprofloxacin in fish-farm water was also accomplished through a ratiometric fluorescence optical fibre sensor. The Y-type optical fibre spectrometer was decorated with Cd-Te quantum dots composite, functionalised with glutathione and mercaptopropionic acid (GMPA@CdTe-QDs) [87]. The sensor showed high selectivity towards interferences, which was attributed to the difference between the fluorescence emission wavelength of ciprofloxacin at 430 nm, and its analogues (500 nm) [87].

## 6. Harmful Algal Bloom and Its Toxins Monitoring

As aforementioned, an overabundance of nitrogenous compounds in water induces the eutrophication of water bodies, increasing the probability of algal blooms [88]. Some of these algae species can produce natural toxins that can be detrimental to the aquatic ecosystems and their fish and shellfish species, as well as socio-economic effects related to damages in recreational/touristic sites. Furthermore, human poisoning outbreaks are attributed to the consumption of toxin-contaminated fish/seafood or drinking water [89].

These toxins are commonly classified according to the symptoms provoked in humans, including amnesic shellfish poisoning (ASP), diarrheic shellfish poisoning (DSP), and paralytic shellfish poisoning (PSP), as well as additional lipophilic toxins such as azaspiracids (AZAs), yessotoxins (YTXs), and pectenotoxins (PTXs) [90]. Current methods of detection for algal biotoxins are primarily established for shellfish where regulations are in place within the European Commission (Regulation (EC) No. 2074/2005 and No. *15/2011*) [91,92,93] and worldwide Codex, STAN 292-2008 [90]. However, no regulations are currently established for detecting biotoxins in aquatic ecosystems. The only toxin with a guideline value is microcystins (MC), recommended by the World Health Organization (WHO) at a concentration of 1 μg L^−1^ in drinking water [94]. Therefore, implementing those limits requires the development of new detection methods for every group of toxins, acting as early warning tools in the screening for water quality monitoring. 

Until recently, mouse bioassays (MBA) were kept as the standard method for detecting algal toxins, which involve intraperitoneal injections of shellfish extracts to mice and monitoring the symptoms and time to death [7]. However, the increasing ethical concerns among the community and technical limitations, such as low sensitivity and inaccuracies due to matrix effects, allowed non-animal methods to gradually replace MBA. As a result, analytical techniques such as HPLC [95] and LC-MS/MS [96] started to be implemented as an alternative to the mouse bioassay and have been adapted for detection in algal and seawater samples.

Furthermore, novel biosensing technologies have emerged based on surface plasmon resonance and planar waveguides for algal toxin analysis. In this sense, the project BioCop was proposed within the European Union (EU) Sixth Framework Programme for Research and Technological Development, focusing on designing an SPR biosensor assay to detect paralytic shellfish poisoning (PSP). The device was developed using a saxitoxin-binding protein and chip surface in tandem [97]. 

Campbell et al. validated an SPR device to detect tetrodotoxin (TTX), obtaining levels of detection as low as 200 μg kg^−1^ and up to 800 measurements per chip. In addition, the assay was validated under AOAC standards for gastropods and puffer fish [98]. Meanwhile, Reverté et al. used planar waveguide cartridges based on an immunoassay scheme to detect TTX [99], seeing levels as low as 0.4–3.29 μg g^−1^ in pufferfish tissue. 

Meanwhile, detecting yessotoxin and brevetoxin-2 toxins was accomplished using desulfur-yessotoxin (dsYTX) as a ligand immobilised onto an SPR chip. In addition, the method proposed an indirect assay in which the toxins in the sample (at different amounts) compete with ligand dsYTX for binding to the phosphodiesterase II (PDEII) in a competitive assay [100]. Finally, in another study, the quantification of yessotoxin was established through a direct assay, where the interaction of YTX with the phosphodiesterase I (PDEI) was measured using the commercial BiaCore X—SPR-based equipment [42]. 

One of the first SPR biosensors for paralytic shellfish toxins (PSTs) was established by Fonfria et al. (2007) [101], employing an inhibition assay between an anti-gonyautoxin 2,3 (GTX2/3) and a saxitoxin-CM5 chip, was performed. The assay allowed the quantification of saxitoxin (STX), decarbamoyl saxitoxin (dcSTX), decarbamoyl gonyautoxin 2,3 (dcGTX2/3), and gonyautoxin 5 (GTX5) at concentrations from 2 to 50 µg L^−1^. The study also tested the interference of mussels, clams, cockles, scallops, and oysters’ matrices. Later, a novel protocol for STX detection was proposed by Chen et al. (2007), where the interaction between the toxin and calix [4] arene derivatives was studied through SPR [43]. The study established that the concentration of STX was proportional to the SPR angle shifts that resulted from molecular interaction between the toxin and the calix [4] arene self-assembled monolayer formed onto the chip surface. This binding interaction occurred through the π–π and van der Waals of the calix [4] arene with STX [43].

Then, Yakes et al. (2011) described an SPR immunoassay for the detection of saxitoxin (STX) in a clam-extract matrix under an inhibition scheme [102]. The toxin was bound to the gold-coated chip, whereas the anti-saxitoxin and the sample solutions (containing the toxin) were mixed. Once the mixture is exposed to the saxitoxin chip, the free antibodies bind to the substrate, and the SPR signal is measured [102]. Later, Haughey et al. established an SPR immunoassay employing polyclonal antibodies (R895) and monoclonal antibodies (GT13A) for saxitoxin, measured using two SPR platforms: a Biacore Q and Biacore T100 system. In the study, sixty shellfish extracts (including mussels, cockles, and scallops) were tested, resulting in the Biacore T100 showing a higher matrix effect attributed to a mismatch between the running buffer and the background from the antibody solution. Finally, the results demonstrated that using the polyclonal antibody has a slightly higher agreement when compared with high-performance liquid chromatography (HPLC) and mouse bioassay (MBA) methods, ranging from 85% to 94.4%. In contrast, monoclonal antibodies range from 77.8% to 100% [103]. Meanwhile, Rawn et al. reported that the use of polyclonal (R895) and monoclonal (GT13A) antibodies in an SPR platform did not respond satisfactorily to the detection of saxitoxin N-1-hydroxylated analogues, such as neosaxitoxin [104]. Saxitoxin (STX) was also examined in shellfish using a surface plasmon resonance (SPR). In the methodology proposed, three different receptors were investigated:  a sodium channel receptor (SCR), a monoclonal antibody (GT13-A), and a polyclonal antibody (R895). The biosensor was based on an inhibition assay involving the immobilisation of STX by amino-coupling reacting against each of the three receptors. The results showed a better response employing the polyclonal antibody R895 (1.56 µg L^−1^) [105].

In 2019, Ha et al. reported the first time using a localised SPR (LSPR) aptasensor to detect STX in buffer and mussel samples, obtaining a limit of detection (LOD) of 2.46 µg L^−1^ and recoveries of 96.13–116.05%. Detecting the toxin was accomplished by integrating a gold nanorod (GNR) and the aptamer, whose conformational structural change due target/aptamer binding event on the GNR surface provokes a refractive index (RI) increase, generating an LSPR shift. Thus, the toxin can be recognised by measuring the LSPR shift [106]. The LSPR sensors use intrinsic electric field waves of novel metal nanoparticles. When molecular-binding events occur near the metallic particles’ surface, a wavelength shift is induced in the absorption spectrum of the particle (LSPR shift). In the study, the STX aptamer developed as a receptor employed an improved method based on the π–π stacking interaction between graphene oxide and single-stranded DNA (ssDNA), showing a higher efficiency and yield [106]. Regarding the LSPR technique, it is also common for its coupling to quantum dots (QDs) as fluorescent labels. However, although various applications involving quantum dots have been reported in aquaculture pollution detection, those uses have been established as chemiluminescent or fluorescent sensors, not as plasmonic quantum sensors (based on the LSPR principle). For instance, Chen et al. (2016) reviewed using QDs as fluorescent probes for detecting heavy metals, pesticide residues, antibiotics, and ammonia [107].

On the other hand, biolayer interferometry (BLI) is another label-free and real-time optical technique that employs fibre-optic to measure the interactions between biomolecules [108]. The binding event between the target molecule and its ligands on the fibre’s surface provokes a shift in the interference spectrum of reflected light (Δλ) [109].

In 2016, Gao et al. [110] employed the aptamer GO18-T-d to construct a BLI aptasensor to detect GTX1/4 in spiked shellfish samples. In the study, the authors reported that the detection signal significantly depended on Mg^2+^ concentration and buffer pH. Later, a competitive biolayer interferometry aptasensor was developed using the aptamer M-30f to detect STX in the shellfish matrix, ribbon fish, and water components with a good recovery of 101.40–107.26% [111].

The detection of domoic acid (DA) toxin has also been reported through an SPR immunosensor and monoclonal antibodies (anti-DA). The gold-coated SPR chips were functionalised employing a novel methodology that explained the use of a long-chain of HS(CH_2_)_11_(C_2_H_5_O)_6_NH_2_ thiol [39] that allowed the direct binding of the toxin by carboxyl groups on DA. Meanwhile, the short-chain HS(CH_2_)_11_(C_2_H_5_O)_4_OH acted as a non-fouling agent to avoid unspecific bindings [112]. On the other hand, methanolic extracts of scallops, mussels, cockles, and oysters were also analysed using a competitive inhibition format to determine the presence of domoic acid (DA) [113]. In the study, rabbit polyclonal antibodies against DA interacted in a mixed solution with the toxin standard/sample extracts and were then allowed to interact with molecules of domoic acid immobilised on the SPR chip surface. The method permitted 800 cycles using a chip without loss of surface activity [113].

In terms of molecularly imprinted polymers (MIP) technology, these synthetic receptors have also been reported for detecting DA under a competitive scheme. The MIP was directly created by photo-grafting onto an SPR chip, obtaining a film of 40 nm where the competitive binding was performed between toxin molecules interacted with horseradish peroxidase-DA conjugates. The method was also evaluated using monoclonal antibodies as natural receptors for the toxin, resulting in a lower detection limit than the technique where an MIP-modified SPR chip was employed (1.8 µg L^−1^ in front of 5 µg L^−1^). In addition, the MIP chip allowed continuous measurement for two months [44].

Okadaic acid (OA) was also detected using polyclonal antibodies toward the toxin conjugates (OA–bovine thyroglobulin and OA–*N*-hydroxy succinimide ester) immobilised onto an amine SPR chip surface. The antibody was diluted 1/750 and mixed in a ratio of 1:1 with the toxin standard. The method was employed to analyse several spiked shellfish samples with OA at 126 ng g^−1^ [114]. Furthermore, fibre-optic-based chemiluminescence was also designed to detect okadaic acid. In the study, OA conjugates were immobilised on polyethersulfone membranes and tested in a competitive assay with free toxin molecules and horseradish peroxidase-labelled monoclonal antibodies. The method showed a stable response during 34 cycles, allowing the detection of OA with a limit of detection of 0.2 g per 100 g of mussel extract [115].

On the other hand, protein G-coated magnetic particles were used to support the immobilisation of antibodies against OA in an SPR device under a competitive scheme. SPR analysis of antibody–magnetic particle conjugates demonstrated up to 11-fold higher SPR signals than free antibodies (direct binding). The conjugates in the direct competition assay provided a 2.6 μg L^−1^ (threefold lower LOD). In the study, a real mussel matrix obtained from the Ebro Delta Bays during a diarrheic shellfish poisoning event was tested, showing no interference in the OA quantification and validated by liquid chromatography–tandem mass spectrometry and a mouse bioassay (MBA) [51].

An SPR direct assay for palytoxin (PLTX) in grouper and clam was performed for kinetic measurements and quantification [116]. The method showed a limit of detection of 2.8 and 1.4 μg L^−1^ in samples containing 10% grouper and 10% clam, respectively. For all experiments, mouse monoclonal antibodies (anti-PLTX) were covalent coupling to the surface’s chip, showing that as the concentration of PLTX in the sample increased, the SPR signal (RU) augmented proportionally due to the direct binding of PLTX to the anti-PLTX. The cross-reactivity was also tested against other marine toxins like saxitoxin, tetrodotoxin, maitotoxin, pectenotoxin, okadaic acid, and dinophysistoxin, showing no competition or additive interaction at concentrations as high as 25 μg L^−1^ [116]. Then, the PLTX was measured in a multiplexing SPR system, along with the simultaneous detection of domoic acid, okadaic acid, and saxitoxin [117]. Although the palytoxin was measured at 1:2 dilution, a critical matrix effect was observed, and difficulties in the study resulted in the validation assay for palytoxin not being performed [117].

Biolayer interferometry based on a horseradish peroxidase-labelled aptamer (PTX-13) as biorecognition receptors were employed to detect the marine biotoxin palytoxin (PTX) in spiked shellfish and seawater. The assay consisted of a competitive binding scheme between the aptamer and the immobilised palytoxin on the biosensor surface and PTX in samples, showing a limit of detection of 0.04 ng L^−1^ [118].

The cyanobacterial growth also produced a toxin called microcystins (MC), commonly excreted in fresh waters by species of the genera *Planktothrix, Microcystis, Aphanizomenon, Nostoc*, and *Anabaena,* but with occurrence in brackish and marine environments [119]. The chemical structure of MCs presents a variable amino acid composition, which is labelled depending on its “X” and “Z” positions (MC-XZ). For example, MC-LR contains the amino acids leucine (L) and arginine (R), respectively. Accordingly to the Guidelines for drinking-water quality published by the World Health Organization (WHO), a concentration of 1 µg L^−1^ of MCs (sum of all congeners, free plus cell-bound) causes health significance [94].

For the detection of MCs, Long et al. (2009) reported a fibre-optical biosensor based on an immunoassay, where MC-ovalbumin conjugates were covalently immobilised onto an optic probe by self-assembled thiol-silane monolayer, followed by the interaction with MC-LR antibodies [120]. The detection limit obtained was 0.03 µg L^−1^ showing high resistance to non-specific protein binding and more than 150 assay cycles without any damage to the surface-immobilised MC-ovalbumin. The immunoassay performance was validated concerning conventional high-performance liquid chromatography (HPLC), showing a correlation of *r*^2^ = 0.9978 [120]. Meanwhile, Herranz et al. in 2010 evaluated the detection of MC-LR in tap water, employing an SPR biosensor based on a competitive inhibition assay, in which the toxin was covalently immobilised onto the chip surface. The method showed a detection limit of 0.073 µg L^−1^ and a working range from 0.2 to 2.0 µg L^−1^. The system allowed four simultaneous determinations in 60 min and up to 40 assay-regeneration cycles (50 mM NaOH). Thus, the cross-reactivity assay showed 88% for microcystin-RR (arginine-arginine) and 94% microcystin-YR (tyrosine-arginine) [121]. Meanwhile, the study of Devlin et al. (2014) reported the detection of MC-LR in an SPR immunoassay. Cross-reactivities of 108% with microcystin-RR (arginine-arginine); 68% in MC-YR (tyrosine-arginine); 69% for MC-LA (leucine-alanine); 71% MC-LW (leucine-tryptophan); 68% MC-LF (leucine- phenylalanine); and 94% nodularin were reported [122]. Later, in 2013, an automated online biosensing platform was applied to detect MC-LR continuously. The system monitored Lake Tai (China) for almost a year, with measurements every six hours and a calibration per day. The measurements consisted of an indirect competitive chip between an MC-ovalbumin conjugate, immobilised in a gold chip, and monoclonal antibodies against MC-LR. The chip was exchanged once a month and the platform exhibited a limit of detection of 0.09 µg L^−1^ [123]. Finally, in 2001, DNA aptamers were designed for the direct detection of MCs, demonstrating a specific binding in the range of 50–1000 mg L^−1^ by surface plasmon resonance (SPR) [124].

Regarding the cylindrospermopsin (CYNs) toxin, Elliott et al. (2013) reported monoclonal and polyclonal antibodies for CYNs. Competitive indirect ELISA and SPR techniques characterised the antibodies, obtaining a sensitivity of 0.027 to 0.131 µg L^−1^ and 4.4 to 11.1 µg L^−1^ for ELISA and SPR, respectively [125].

In terms of multiplexing assays, McNamee et al. developed a multiplexing test capable of detecting up to five different marine and freshwater toxins (STX, DA, OA, MC-LR, and cylindrospermopsin) on a single planar waveguide cartridge. The test employed antibodies that recognise the toxin conjugates in a competition-based assay in algal and seawater samples within 15 min [126]. Then, another multiplex SPR was used to simultaneously detect paralytic shellfish poisoning (PSP) toxins, okadaic acid (and analogues), and domoic acid, but now in algal and seawater samples collected from Spain and Ireland. This method showed detection limits of 0.82, 0.36, and 1.66 µg L^−1^ for PSP, okadaic acid, and domoic acid, respectively [127].

Campbell et al. (2011) proposed a multiplexing system (multi SPR) for the analysis of marine biotoxins from different groups: domoic acid, okadaic acid, and paralytic shellfish toxins [128]. The parent compounds of the toxins were immobilised within a single chip, allowing the compartmentalisation of different binding reactions at once to distinguish between toxin families [128]. Later, McCoy et al. (2014) employed this multi-SPR technology to monitor blooms of *Alexandrium minutum* occurring annually in the north channel of Cork Harbour (south coast of Ireland). In particular, the study was centred on the summer months of 2011. The toxin produced by dinoflagellates was detected in sweater by SPR and compared to three other techniques: Microarrays for the Detection of Toxic Algae (MIDTAL), commercial enzyme-linked immunosorbent assay (ELISA), and high-performance liquid chromatography (HPLC) [129]. The microarray signals and SPR biosensor followed a trend with light microscopy results, and both techniques indicated detection limits below 4000 cells of *A. minutum* in natural seawater samples [129]. Another study for detecting neurotoxic paralytic shellfish toxins produced by genera *Alexandrium* was reported but using an innovative planar waveguide device, integrated by a transparent substrate containing an array of toxin–protein conjugates and assembled in a cartridge to allow the injection of samples [130]. The recognition was based on a competitive assay format with high-affinity antibodies to paralytic shellfish toxins and labelled secondary antibodies capable of generating fluorescent signals. The limit of detection was 12 ng L^−1^ in the sweater, being able to detect the toxin at an algae cell density of 10 cells L^−1^ [130].

With this regard, a trend toward developing plasmonic sensors for detecting toxins can be observed. However, as aforementioned, there are various areas along aquaculture operations where plasmonic sensors can intervene to improve the rapid assessment of the quality of the seafood. Furthermore, fishery products are important from a nutritional perspective and an item to foreign exchange and international trade. Therefore, quality maintenance is of utmost importance in production and over the supply chain on its commercialisation.

## 7. Fish and Shellfish Freshness: Safety Evaluation

Fish and shellfish products are highly perishable and prone to variations in quality due to temperature variations, cross-contamination during food handling, or simply improper storage, affecting the taste and their safety [131]. Thus, fish and seafood quality is essential for consumers and the food industry, requiring non-destructive, rapid, and reliable methods to detect food safety in real-time.

In this sense, biogenic amines (BAs) are small organic molecules that result from enzymatic decarboxylation of amino acids or amination in rich-protein food. Biogenic amines increase under improper storage to be employed as food spoilage [132]. The most common BAs are putrescine, cadaverine, spermidine, and spermine, and they are generally toxic to humans. The presence of volatile amines, such as ammonia, dimethylamine (DMA), and trimethylamine (TMA), is also typical, producing off-odours and diminishing the organoleptic quality of the products [132].

This context improved the development of LSPR sensing methods for gas detection, incorporating nanoparticles modified with functional layers to facilitate preferential adsorption of gaseous analytes, which generally induce refractive index changes (LSPR in reflection mode). For example, Tseng et al. (2017) reported the development of a paper-based plasmonic refractometric sensor to monitor BAs generated from spoiled fish [133]. The hollow Au−Ag alloyed (HGNs) was embedded using a reversal nanoimprint lithography (rNIL). In the study, the HGNs exhibited a wavelength shift upon the adsorption of the putrescine-spiked salmon samples, showing a limit of detection of 13.8 mg L^−1^. On the other hand, no modification was observed under the exposure to N_2_, CO_2_, and water vapour, highlighting the excellent selectivity of the method [133].

Plasmonic colourimetric sensors based on noble metal nanoparticles have also been explored to detect volatile amines, taking advantage of the LSPR’s unique properties as a function of particle aggregation or etching (colourimetric sensing). For instance, Heli et al. [134] established the ammonia gas detection by silver nanoparticles (AgNPs) embedded in a bacterial cellulose nanopaper, showing a change in colour from amber to grey or taupe upon exposure to spoiled fish, which was attributed to the decrease of the AgNPs population by ammonia corrosion. A limit of detection of 28.7 μg of ammonia volatilised from 50 mL was obtained by the plasmonic nanopaper [134]. Other sensor platforms for visual monitoring food spoilage based on plasmonic colourimetric gold nanoparticles have been reported for ammonia [135] and dimethyl sulfide and histamine for fish, crustaceans, and preserved meat [136].

**Figure 4:**
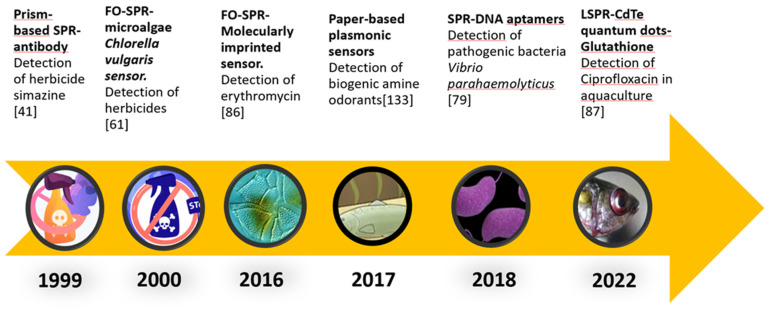
Roadmap of the implementation of plasmonic techniques and different recognition elements for their use in aquaculture.

Figure 4 shows a roadmap describing the evolution of the implementation of different recognition elements in plasmonics systems assisting some of the aquaculture areas revised in this study.

In general, plasmonic techniques require small amounts of non-toxic reagents, such as buffer solutions based on salts (phosphates, acetates, and chlorides), and the analysis of water samples requires a simple filtration before measurements [42]. In the case of fish or shellfish samples, a previous extraction step is needed with solvents that are considered toxic, such as alcohols, chloroform, or acidic solutions [106]. However, the extraction procedure occurs outside the fish farming facilities, avoiding contact with the products. Furthermore, these are official extraction procedures and do not represent higher risks than conventional techniques [106]. Regarding nanoparticles and immobilised molecules used as recognition elements, the challenge is to achieve a sufficiently strong binding so that the receptor does not detach. It has been demonstrated that the immobilisation of these recognition elements enzymes maintains their integrity during multiple cycles of measurements [137]. Moreover, the recognition elements revised in this study have been indicated as biocompatible and non-toxic, including the quantum dots [138]. For further understanding, Table 2 summarises the analytical parameters reported in the revised articles for plasmonic sensors in aquaculture applications.

## 8. Final Remarks and Perspectives

Through this study, the importance that the surface plasmon resonance technique has acquired for the different quality control processes that are carried out throughout the production chain and post-production activities performed by aquaculture has been highlighted. In fact, the tendency observed in this economic activity is clearly directed towards the development of plasmonic sensors for the detection of toxins generated by algal blooms. However, it is worth noting that few studies have pioneered research on sensing stress indicators, detecting pathogens (viral and bacterial), and freshness assessment of seafood. In addition, the lack of studies on applications of plasmonic sensing other than fish, such as oysters, shrimp, or crustaceans, denotes potential applications to be developed in the near future.

One of the reasons implementing the plasmonic sensor in farming facilities (tanks or ponds) in aquaculture has been very successful is because the optical fibre probes offer an operational advantage regarding water monitoring. This is because they are readily introduced into the tanks and operate remotely, making this type of sensor very suitable for aquaculture. Meanwhile, the tendency to use prism-based SPR to detect toxins and the LSPR technique for monitoring biogenic amines as a freshness indicator can be highlighted.

Optical fibre probes still have limitations since successful surface modification and reliable immobilisation of recognition elements are required to improve detection efficiency and decrease non-specific binding. In particular, the sensitivity of LSPR sensors is negatively affected by surface environment alterations, causing disturbances in the shape of the SPR peak. Nevertheless, the most significant limitation still regards sample preparation and processing since the presence of other components or contaminants in real matrices (mainly in food products) always influences the analytical performance of sensors, which deters their on-site application. The aforementioned is reflected in the number of biosensors tested in buffer or water and only a few on spiked samples. Furthermore, the chemical particularities of the target compounds lead to specific sample extraction and detection, making the analysis specific for individual analytes or families of similar compounds, representing an important challenge in effective detection.

An alternative to overcome the limitation of detection in complex samples is using an array of sensors that respond to a broad spectrum of analytes instead of a specific one. Thus, the collective data could create fingerprint patterns associated with the sample characteristics. This approach has been explored in other areas, with the development of optoelectronic noses assisted by artificial intelligence or neural networks. On the other hand, some efforts have been focusing on SPR multiplexing technology, with the development of multichannel devices, which could resolve some of the difficulties in specificity performance. A few approaches to multiplexing studies in aquaculture have been detailed in the present review.

Moreover, future improvements regarding aptamer-based plasmonic biosensors should be interesting since most of the works summarised have focused on detecting toxins generated by harmful algal blooms. Further, the miniaturisation and integration of a sample collection system are required. In this sense, microfluidic systems, which are already reported in other fields, could be implemented to enable automated sample handling. Thus, a fully integrated biosensor would significantly contribute to implementing a real lab-on-a-chip in the aquaculture industry. Regarding miniaturisation, it is worth noting that paper has recently been rediscovered as low-cost material in sensing platforms providing flexibility, thinness, and light weight, unlike glass material, which is commonly deposited on metallic films to generate plasmonic phenomena. Future research based on plasmonics techniques should address more studies regarding the quantum plasmonic principle as an approach to enhance the sensitivity of plasmon-coupling-based sensors. Although some plasmonic sensors have been demonstrated to exhibit single-molecule sensitivity, complicated fabrication procedures or sophisticated instruments prevent their practical application. Thus, more efforts should be made to simplify the sensing application’s transducers since, nowadays, the detection still requires sophisticated devices in outdoor environments, which may not be available.

Furthermore, plasmonic colourimetric sensors based on noble metal nanoparticles are an excellent alternative to satisfy this purpose, allowing measurement to be carried out by the naked eye. In addition, continuous efforts to obtain the lowest detection limit should be maintained. For instance, using aptamers coupled to a sandwich-assay format enhances the detection at concentrations in order of magnitude of picomolar (pM).

Finally, while emerging plasmonic technologies are sure to have long-lasting impacts, they need to become more reliable and readily deployed. Meanwhile, the existing operational process in aquaculture needs to evolve as well; in this way, a robust platform can be laid in the end to balance cost and ease of implementation.

## Figures and Tables

**Table 1:** Comparison of latest sensing technologies used in aquaculture.

Sensing Technology	Advantages	Disadvantages	References
Prism-based SPR	Allows the study of binding interactions in a label-free format (i.e., no addition of fluorescent tags is necessary). Highly sensitive to the refractive index (RI) of the medium in contact with the metal film (usually aqueous solution for aquaculture applications). Widely established and commercially available. SPR studies can exist in a multiplexed employing multichannel device.	The prism can be bulky and difficult to incorporate into miniaturised platforms. Only detects RI changes close to the metal film surface (extends ~200 nm). Temperature control is needed to produce stable SPR signals. The sensing device cannot be used for remote sensing applications.	[10]
LSPR	More amenable to multiplexing and miniaturisation than prism-based SPR. Detection systems can be tuned by varying the nanoparticles’ size, shape, and composition. Allows the use of wavelengths that do not overlap with the spectral features of strongly absorbing samples (natural chromophores). The plasmon resonant nanostructures can be used as fluorophore tags LSPR sensors are susceptible to the RI of the surrounding medium.	Detects RI changes that happen only tens of nanometers into the surrounding medium. LSPR sensors have dramatically reduced sensing volumes, extending the detection limit to the single-molecule level. Sensing experiments need to ensure that the binding of the target molecule happens within the sensing volume as opposed to outside of it, especially when it involves bulky molecules.	[11]
FO-SPR	SPR probe can be miniaturised. Flexible, can be easily moved, and allow remote sensing application. Temperature control is not needed to produce stable signals. Multiplex analysis can be allowed by the guiding light in different wavelengths simultaneously	Complex fabrication and surface functionalisation. Damage of sensing elements due to prolonged exposure to incident light. Slow response time due to the diffusion effect of analytes.	[12]
Electrochemical sensors	Low-cost production of electrodes and microelectronic circuits. The straightforward interface of electronic read-out and processing. Multiple enzymatic labels increase the signal per event.	There are electrical safety hazards and electrical interference. Factors such as pH and ionic strength in fluids can r significantly affect the sensor’s response. The miniaturisation of electrochemical sensors tends to increase the signal-to-noise. These devices use redox molecules that mediate the electrochemical reaction at the working electrode. The lifetime of electrodes diminishes due to fouling effects.	[13]
Quantum dots sensing	Excellent fluorophores, resistant to thermal and photochemical reactions. Simple manufacturing process	Low fluorescence quantum yield Requires surface passivation process (coating). Sensitivity relies on the recognition element.	[14,15]
Polymerase chain reaction (PCR)	Highly sensitive, accurate, and good repeatability. Real-time analysis.	Require PCR instrument. Costly reagents. Time-consuming. Requires technical expertise.	[16,17]
Chromatography–mass spectrometry	Highly sensitive, accurate, and good repeatability.	Costly reagents. Time-consuming. Requires technical expertise. Chromatography cannot meet the requirements for in-field detection.	[7]

**Table 2:** Plasmonic approaches for sensing applications in aquaculture.

Analyte	Plasmonic Method	Recognition Element	Analytical Parameters	Reference
Nitrite	LSPR	Satellite-like AuNPs	The linear of 0–1.0 mg mL^−1,^ and the detection limit of 3.0 µg L^−1^	[55]
Ammonia	Oxazine-FOSPR	Oxazine 170 perchlorate	Limit of detection 1.4 mg L^−1^	[57]
Ammonia gas	FO-SPR	Oxazine 170 perchlorate	Limit of detection mg L^−1^	[59]
Ammonia	FO-SPR	Oxazine 170 perchlorate	Working range of 100 to 900 µg L^−1^. Sensitivity of 0.0036 mg L^−1^	[60]
Herbicides	FO-SPR	Microalgae *Chlorella vulgaris*	Limit of detection: 5nM for atrazine, 1 nm for simazine, 0.1 nM for diuron, 5 µM for alachlor, 0.1 mM for glyphosate	[61]
Atrazine	FO-SPR	Molecularly imprinted polymers	Concentration range of 0 M–10−7 M. Sensitivity: 10−12 M	[67]
Irgarol 1051	Interferometric	Antibody	Limit of detection 3 ng L^−1^	[40]
Irgarol 1051 and tetracycline	Interferometric	Antibody	Limit of detection of 0.04 µg L^−1^ and dynamic range from 0.08-0.5 µg L^−1^ for tetracycline Limit of detection of 0.07 µg L^−1^ with a dynamic range from 0.2-12 µg L^−1^ Irgarol	[68]
Simazine	SPR	Antibody IgG antibodies and FAB fragments	Limit of detection of 0.11 µg L^−1^	[41]
Carbaryl	SPR	Antibody	Working range of 2.78–3.55 µgL^−1^	[48]
Creatinine	FO-SPR	Enzyme creatinase	A sensitivity and limit of detection of 3.1 µM and 86.12 µM, respectively. Linear range of 0-2000 µM	[49]
Cortisol	FO-SPR	Antibody	Working range of 0.01 to 100 µg L^−1^ with a limit of detection of 1.46 µg L^−1^	[73]
Cortisol	SPR	Antibody	Limit of detection of 1.0 µg L^−1^ (3.6 nM)	[74]
Pathogenic bacteria *Vibrio parahaemolyticus*	SPR	DNA aptamers	Analysing concentrations of ss DNA from 680.1 ng µL^−1^ to 1196.6 ng µL^−1^ with efficiency from 92.98 to 98.15%	[79]
Nervous necrosis virus	FO-SPR	Gold nanoparticles	Limit of detection of 100 µg L^−1^	[80]
Ciprofloxacin	SPR	Molecularly imprinted polymers	Limit of detection 7.1 µg L^−1^	[84]
Erythromycin	Surface plasmon resonance nanosensor	Molecularly imprinted nanoparticles	The linearity range and limit of detection were 0.99 and 0.29 mg L^−1^, respectively	[85]
Erythromycin	FO-SPR	Molecularly imprinted polymers	Working range 0 to 50 µM. Its sensitivity was 5.32 nm µM^−1^	[86]
Ciprofloxacin	FO-SPR	Functionalised glutathione and mercaptopropionic acid nanoparticles	Working range from 0 to 45 µM with a detection limit of 0.90 µM	[87]
Paralytic shellfish poisoning toxins	SPR	Antibody	Limit of detection 120 μg kg^−1^	[97]
Domoic acid (DA) and okadaic acid (OA), saxitoxin (STX), cylindrospermopsin (CYN), and microcystins (MC)	FO-SPR	Antibody	Limit of detection was 0.37 for DA, 0.44 for OA, 0.05 for STX, 0.08 for CYN, and 0.40 ng mL^−1^ for MC	[126]
Tetrodotoxin	SPR	Antibody	Limit of detection 200 μg kg^−1^	[98]
Yesotoxin	SPR	Phosphodiesterase enzymes	Concentrations from concentrations 3 to 12µM. R = 0.9669	[42]
Domoic acid (DA), okadaic acid (OA), neosaxitoxin (NEO) and saxitoxin (SAX)	SPR	Antibody	Workings range of 1.0–6.4, 1.7–14.4, 1.1–6.0, and 1.0–3.7 ng mL^−1^ for DA, OA, NEO, and SAX, respectively	[128]
Okadaic acid	FO-SPR	Antibody	Limit of detection of 0.2 μg per 100 g	[115]
Okadaic acid	SPR	Antibody	Limit of detection 2.6 μg L^−1^	[51]
Saxitoxin	SPR	Calix [4] arene derivative monolayers	Working range of 1.0 × 10^−9^–1.0 × 10^−5^ M	[43]
Saxitoxin	SPR	Antibody	Working range from 0 to 400 ng mL^−1^	[102]
Saxitoxin	LSPR	Aptamer	Limit of detection of 2.46 µg L^−1^	[106]
Microcystin-LR	FO-SPR	Antibody	Limit of detection of 0.03 μg L^−1^	[120]
Microcystins	SPR	Antibody	Limit of detection of 73 ± 8 ng L^−1^	[121]
Cylindrospermopsin	SPR	Antibody	Sensitivity of 4.4 to 11.1 ng mL^−1^	[125]
Putrescine	LSPR	Hollow Au−Ag nanoparticles	limit of detection of 13.8 mg L^−1^	[133]

## Data Availability

No new data were created or analyzed in this study.
